# Circulating Glycated Albumin and Glomerular Anionic Charges

**DOI:** 10.1155/EDR.2003.83

**Published:** 2003

**Authors:** I. Londoño, D. Gingras, M. Bendayan

**Affiliations:** 1 Department of Pathology and Cell Biology Université de Montréal C.P. 6128 Succ. “Centre-ville,” Montreal QC H3C 3J7 Canada

## Abstract

Aiming to discern the mechanisms by which circulating glycated albumin alters the glomerular filtration properties that lead to glomerular dysfunction in diabetes, the authors studied the distribution and densities of anionic charges through the rat glomerular wall upon intravascular infusion of Amadori products, as well as in various conditions of increased glomerular permselectivity. Polylysine-gold was used as the probe to reveal the anionic charges. The study was carried on renal tissue sections of bovine serum albumin (BSA)- and glycated BSA–injected, normoglycemic animals. Results were generated through morphometrical evaluations of the gold labeling. Changes in glomerular anionic distribution were corroborated on renal tissue sections of short- and long-term diabetic rats and of normal newborn rats, situations known for abnormal glomerular filtration. Altered renal function in these conditions was clearly associated with changes in glomerular anionic charges. On the other hand, the infusion of glycated albumin in the circulation of normal rats, though altering glomerular filtration properties, did not modify the distribution and density of the polylysine-gold labeling through the glomerular basement membrane. Thus, anionic charges seem not to be the factor involved in the early changes of glomerular permeability induced by circulating glycated albumin.


*Experimental Diab. Res.*, 4:83–92, 2003
Copyright © Taylor and Francis Inc.
ISSN: 1543-8600 print / 1543-8619 online
DOI: 10.1080/15438600390220025

### Circulating Glycated Albumin and Glomerular Anionic Charges

**I. Londoño, D. Gingras, and M. Bendayan**

*Department of Pathology and Cell Biology, Université de Montréal, Montreal, Quebec, Canada*

**Aiming to discern the mechanisms by which circulating glycated albumin alters the glomerular filtration properties that lead to glomerular dysfunction in diabetes, the authors studied the distribution and densities of anionic charges through the rat glomerular wall upon intravascular infusion of Amadori products, as well as in various conditions of increased glomerular permselectivity. Polylysine-gold was used as the probe to reveal the anionic charges. The study was carried on renal tissue sections of bovine serum albumin (BSA)- and glycated BSA–injected, normoglycemic animals. Results were generated through morphometrical evaluations of the gold labeling. Changes in glomerular anionic distribution were corroborated on renal tissue sections of short- and long-term diabetic rats and of normal newborn rats, situations known for abnormal glomerular filtration. Altered renal function in these conditions was clearly associated with changes in glomerular anionic charges. On the other hand, the infusion of glycated albumin in the circulation of normal rats, though altering glomerular filtration properties, did not modify the distribution and density of the polylysine-gold labeling through the glomerular basement membrane. Thus, anionic charges seem not to be the factor involved in the early changes of glomerular permeability induced by circulating glycated albumin.**

**Keywords** Amadori; Charge Permselectivity; GBM; Polylysine-Gold

This work was supported by funds from the Canadian Institutes for Health Research.

Address correspondence to Moise Bendayan, Department of Pathology and Cell Biology, Université de Montréal, C.P. 6128, Succ. "Centre-ville," Montreal, QC, Canada H3C 3J7. E-mail: moise.bendayan@umontreal.ca

The glomerular basement membrane (GBM) restricts the passage of plasma proteins according to their size, charge, and molecular conformation [1, 2]. Several studies have proposed that the lamina densa [3–5] and/or the slit diaphragms between podocytes [6, 7] could be responsible for the size-restrictive properties of the barrier. The restriction by charges in the glomerular wall has been attributed, on the other hand, to the presence in the basement membrane of anionic structural components, such as the sulphated glycosaminoglycans in heparan sulphate proteoglycans (HSPGs), reviewed in [8], and the carboxyl groups in glycosaminoglycans and sialoproteins in the GBM and the epithelial glycocalyx [9, 10].

In previous studies, we have reported that glycated albumin, once in circulation, penetrates the glomerular wall of normal animals deeper than nonglycated albumin and reaches the glomerular urinary space [11]. Moreover, even in normal animals, glycated albumin alters the glomerular filtration properties of other serum proteins [11, 12]. These alterations encountered in normal animals were found to be transient and lasted up to 48 hours after the introduction of glycated albumin in circulation [12]. No particular accumulation of the tracer was observed in the GBM or the mesangial matrix of infused animals [11]. The mechanisms by which glycated albumin modifies the glomerular filtration properties can be multiple, among others, glycated albumin could affect the anionic charges of the glomerular wall and/or the size of GBM pores. Indeed, changes in anionic charges in the GBM have been reported as likely responsible for the protein leakage and proteinuria occurring during diabetes [13–17]. Isogai and colleagues [4] have demonstrated that loss of glomerular anionic charges in experimental animals could be detected as early as 2 weeks after streptozotocin (STZ)-induced hyperglycemia. Along this line, increased glomerular permeability to endogenous albumin was reported to take place upon 10 days of hyperglycemia, and this without any evident changes in the structure of the GBM [18]. Persistent hyperglycemia is responsible for early glycation of circulating and structural proteins (Amadori adducts) [10, 19], which are abundant in early diabetes [20]. The participation of these modified proteins and their very active reaction products or advanced glycated end products (AGEs) in the initiation and progression of diabetic nephropathies has already been demonstrated [20–22]. It has previously been shown that glycated albumin is a major trigger in the onset of glomerular dysfunction and proteinuria occurring in diabetic condition [11, 12, 23, 24].

Because glycated albumin is able to alter the glomerular permeability [11, 12], we decided to investigate if circulating glycated albumin alters the glomerular permselectivity properties by modifying the density and distribution of anionic charges across the GBM. Evaluation of anionic charges in the glomerular wall has been previously addressed by means of cationic probes, such as ruthenium red, alcian blue, colloidal iron, polylysine, and polyethyleneimine, combined with specific enzymatic treatments [16, 25–29]. These studies have allowed for the localization and identification of anionic charges in the GBM and revealed changes occurring in different pathological situations [25–28]. We have chosen to work with the polylysine-gold probe [30], which has been shown to efficiently reveal anionic charges over the glomerular wall, with high resolution, in normal as well as in experimental and pathological conditions [31]. Being particulate, this probe allows for quantitative determinations of densities and spatial distribution of the labeling [32], which is particularly useful in establishing changes occurring in experimental conditions.

To corroborate the effectiveness of the polylysine-gold probe and better understand the involvement of anionic charges into the overall establishment of glomerular permselectivity properties in the GBM, we applied the same polylysine-gold approach to 2 additional conditions displaying altered glomerular function: (1) during development, at a time point where the permeability properties of the glomerular wall have yet to be established; and (2) in short- and long-term diabetes, in which glomerular filtration properties undergo progressive alterations.

Results obtained indicate that although alterations in glomerular filtration properties parallel changes in anionic charges distribution and density during development and diabetes, infusion of glycated albumin, though modifying filtration properties, seems not to affect glomerular anionic charges.

### MATERIALS AND METHODS

### Animals

Adult male Sprague-Dawley rats weighing 100 to 150 g at the beginning of the experiment were used. In addition, 4-day-old rats were also included in the study. Diabetes was induced in adult rats by a single intraperitoneal injection of STZ, 70 mg/kg body weight, freshly dissolved in 10 mmol/L sodium citrate buffer, pH 4.5. All rats were maintained on standard chow diet and tap water ad libitum for the length of the experiment. No insulin treatment was required. Body weight and glycosuria were weekly recorded. Blood glucose levels were also determined on tail blood samples at least once a month and at time of sacrifice.

### Experimental Protocol

Animals were separated in 7 groups:

(a) Normal adult rats (n = 3) injected intravenously with 50 mg of glycated bovine serum albumin (BSA) [11], a modified form of circulating albumin predominant in early diabetic subjects [21]. The glycated BSA was prepared according to previously described procedures [11].
(b) Normal adult rats (n = 3) injected intravenously with 50 mg of BSA.
(c) Hyperglycemic ($26.7 \pm 1.2$ mmol/L) young adult rats (n = 3), 10 days after STZ injection, which have demonstrated increased glomerular permeability [18] and high levels of circulating glycated albumin [21].
(d) Age-matched normoglycemic controls (n = 3).
(e) Long-term hyperglycemic ($31.2 \pm 1.2$ mmol/L) adult rats (n = 3), 8 to 12 months after STZ injection, presenting the characteristic features of diabetic nephropathy (glomerular hypertrophy, thickened basement membranes, polyuria, and proteinuria).
(f) Age-matched (12 months old) normoglycemic controls (n = 3).
(g) Normoglycemic animals (n = 3), 4 days old, stage at which GBM development is not completed and glomerular permselectivity properties have not yet been established [33, 34].

For tissue sampling, animals were anesthetized with an intraperitoneal injection of urethane and the abdominal cavity was opened. The fixative, 1% glutaraldehyde in 0.1 mol/L phosphate buffer, pH 7.2, was flooded into the abdominal cavity to begin the fixation in situ. After 10 minutes, a slice of renal cortex was sampled, cut in small pieces, and immersed in the same fixative for 2 hours at room temperature. Tissues were then dehydrated in cold graded methanol and embedded in Lowicryl at −20°C. For the animals in groups a and b, the albumin was injected in the cava vein and kept in circulation for 30 minutes [11] prior to fixation and processing as described above.

### Cytochemical Labeling

In order to reveal the anionic sites in the glomerular wall, labeling with a polylysine-gold complex was performed and the results evaluated by morphometrical determinations. Poly-L-lysine (350,000 molecular weight; Sigma, Toronto, ON) was tagged to 10- or 15-nm gold particles, as previously described [30, 31]. Labeling was performed on ultrathin renal tissue sections by floating them on a drop of 150 mmol/L glycine in phosphate-buffered saline (PBS), pH 7.2, for 15 minutes, then on a drop of polylysine-gold diluted in PBS, pH 7.2, or in KCl, 50 mmol/L pH 2.0, for 30 minutes. The grids were washed with distilled water, dried, and stained with uranyl acetate. In order to identify the glomerular wall components contributing to polylysine-gold labeling, enzymatic digestions with neuraminidase (from Clostridium perfringes, 1 U/mL; Sigma) or heparitinase (from Flavobacterium heparinum, 130 U/mL; Sigma), was performed for 1 hour at 37°C [31], prior to the labeling protocol. As a further control, grids were preincubated with untagged polylysine for 30 minutes before performing the labeling protocol.

Morphometrical evaluation of the labeling was carried out with a Zeiss Videoplan image analysis system (Carl Zeiss, Don Mills, ON). For labeling distribution, distances between each gold particle and the endothelial abluminal plasma membrane ($A$) and between the endothelial and the epithelial plasma membranes ($B$) were recorded; the ratio ($R = A/B$) was calculated. These ratio values varied between 0 (gold particle located adjacent to the endothelial plasma membrane) and 1 (gold particle adjacent to the epithelial plasma membrane). Results are presented as histograms and the mean ratio value for each group was calculated. Densities of polylysine-gold labeling were determined by direct planimetry and gold particle counting and expressed as number of gold particles per square micrometer. Only areas displaying transversal sections of the glomerular wall, clearly showing slit diaphragms between podocytes, were selected for the study. The evaluation was performed on digitalized images (recorded on video) at a final magnification of $\times$154,000. The total area evaluated for labeling densities was about 30 $\mu m^2$ for each animal. Concerning labeling distributions through the GBM, between 700 and 1000 gold particles were recorded for each animal. These data were derived from 3 different glomeruli per animal. Statistical assessments for the distributions were performed using the nonparametrical Mann-Whitney and the Kolmogorov-Smirnov tests. Densities of labeling were compared by the Student's $t$ test.

### RESULTS

Upon incubating renal tissue sections of normal animals with polylysine-gold at pH 2.0 (Figure 1*b*), labeling by gold particles was obtained over plasma membranes as well as in some intracellular compartments of almost all renal cell types, particularly in endothelial, distal, and collecting tubular epithelial cells. All basement membranes (glomerular, mesangial, and tubular basement membranes) were heavily labeled. In the glomerulus, the luminal surface of the visceral epithelial cells or podocytes and the luminal surface of endothelial cells were delineated by gold particles, whereas the plasma membranes of mesangial cells showed no or sparse labeling. The GBM was labeled by gold particles, which were rather concentrated on the epithelial side.

The efficiency and specificity of the labeling obtained with the polylysine-gold probe were assessed through various experimental conditions. Applying the polylysine-gold at pH 7.2 led to a rather sporadic staining at the level of the plasma membranes, the cell cytoplasm, and the basement membranes (Figure 1*a*). These results resemble those reported under the same labeling conditions by Goode and colleagues [10]. Applying the polylysine-gold at pH 2.0, as reported previously [31], the labeling displayed a more restricted distribution, with selective intense decoration of the plasma membranes (Figure 1*b*). The cell cytoplasm remained rather free of labeling. The GBM was intensely labeled with a concentration of the gold particles towards the lamina externa (subepithelial side) (Figure 1*b*). The treatment of tissue sections with heparitinase prior to labeling with polylysine-gold led to the extraction of labeling sites of the GBM (Figure 1*c*), indicating that polylysine-gold binds to heparan sulphate proteoglycans. Conversely, treating the tissue sections with neuraminidase removed all plasma membrane labeling (Figure 1*d*), indicating that polylysine-gold binds to sialic acid residues at this location. GBMs remained labeled after neuraminidase treatment, suggesting a minimal contribution of sialic acid–containing glycoproteins to the anionic charges of the GBM. Finally, by pretreating the tissue sections with polylysine prior to labeling with the polylysine-gold complex abolished all signals (Figure 1*e*), further supporting the specificity of the results. Thus, and as reported previously [31], we proceeded working at pH 2.0.

Normoglycemic animals injected with BSA (Figure 2) as well as those injected with glycated BSA (Figure 3) showed similar polylysine-gold labelings in glomerular cell plasma membranes and in the GBM, with high intensities in the subepithelial side. Upon morphometrical evaluations, GBM labeling distribution histograms obtained for these 2 groups of animals were quite similar. In both cases, the GBM labeling displayed an asymmetrical right-sided distribution (Figures 2*b* and 3*b*). Mean $R$ values were not significantly different ($0.77 \pm 0.01$ for the BSA-injected versus $0.76 \pm 0.01$ for the glycated BSA–injected animals). GBM labeling densities were also similar for both groups ($188 \pm 4$ gold particles/$\mu m^2$ for the BSA-injected versus $182 \pm 6$ gold particles/$\mu m^2$ for the glycated BSA–injected rats). Tissues from normal noninjected adult rats (Figure 4) displayed GBM labeling patterns similar to those obtained on tissues of BSA-injected animals. The GBM labeling

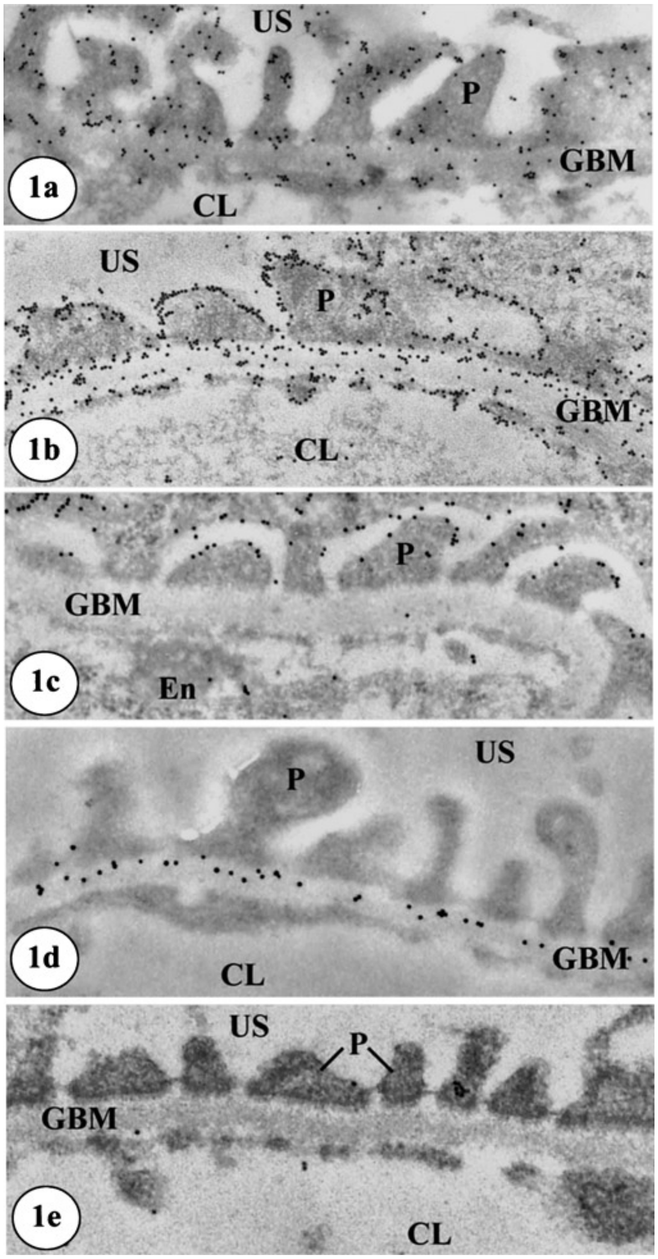

**FIGURE 1**
Polylysine-gold labeling on the glomerular wall of normal rats. (*a*) Polylysine-gold in PBS, pH 7.2. Gold particles are sporadically located in the cytoplasm of epithelial podocytes (P) and endothelial cells as well as through the glomerular basement membrane (GBM). Polylysine-gold, 10 nm. (*b*) Polylysine-gold in KCl, pH 2.0. An intense labeling by gold particles decorates the P plasma membrane. It is also abundant on the epithelial side of the GBM and sparse on the subendothelial side. Polylysine-gold, 10 nm. (*c*) Preincubation with heparitinase followed by polylysine-gold complex, pH 2.0. Labeling is restricted to the (P) plasma membrane. Only very few gold particles are found on the GBM. Polylysine-gold, 15 nm.

distribution histograms showed a prominent peak on the right with a mean $R$ value of $0.76 \pm 0.01$ (Figure 3*b*). GBM labeling densities were, however, slightly lower ($159 \pm 6$ gold particles/$\mu m^2$). Thus, the presence of BSA or glycated BSA in circulation seems not to affect the pattern of distribution of the anionic charges across the GBM.

For the short-term hyperglycemic rats (10 days after STZ injection), which have demonstrated alterations in glomerular permselectivity [18], the polylysine-gold labeling was also located on the subepithelial side of the GBM (Figure 5), though occupying a wider region than that of the age-matched control rats (Figure 4). In fact, the histograms revealed a shift of the labeling towards the center of the GBM (Figures 4*b* and 5*b*). Mean $R$ values for these 2 groups of animals differed significantly ($0.64 \pm 0.01$ for the hyperglycemic animals versus $0.76 \pm 0.01$ for the age-matched controls; $P < .05$). GBM labeling densities were in the same range, though slightly higher for the hyperglycemic young animals ($178 \pm 6$ gold particles/$\mu m^2$ versus $159 \pm 6$ gold particles/$\mu m^2$; $P < .05$).

Long-term hyperglycemic animals (8 to 12 months after STZ injection) display characteristic diabetic nephropathy with thickening of the GBM, increased glomerular permeability, and proteinuria [1]. The labeling distribution in the GBM of these diabetic rats was significantly altered compared to their age-matched controls (Figures 6 and 7). Gold particles were distributed throughout the GBM (Figure 7). The corresponding GBM labeling histograms displayed a rather homogenous distribution (Figure 7*b*), which contrasts with the asymmetric distribution of the labeling obtained for the age-matched control group (Figure 6*b*). Mean ratio ($R$) value for the diabetic animals was significantly lower than that of their age-matched control animals ($0.60 \pm 0.01$ versus $0.73 \pm 0.01$; $P < .05$). On the other hand, GBM labeling densities of diabetic animals were significantly lower compared to their age-matched control ($66 \pm 2$ gold particles/$\mu m^2$ versus $81 \pm 3$ gold particles/$\mu m^2$). Interestingly, GBM labeling densities among normoglycemic animals vary along with age, with lower values for older animals ($81 \pm 3$ versus $159 \pm 6$ gold particles/$\mu m^2$).

In immature glomeruli of the 4-day-old rats, characterized by nonfused basement membranes, labeling was located

←

(*d*) Preincubation with neuraminidase followed by polylysine-gold complex, pH 2.0. Gold labeling is restricted to the GBM, the (P) plasma membrane being completely negative. Polylysine-gold, 15 nm. (*e*) Preincubation with untagged polylysine followed by polylysine-gold complex, pH 2.0. Labeling is completely abolished in all locations. Polylysine-gold, 15 nm. CL, capillary lumen; En, endothelial cell; P, podocyte; US, urinary space. Magnifications: ×30,000 (*a*); ×28,000 (*b*, *e*); ×32,000 (*c*); ×26,000 (*d*).

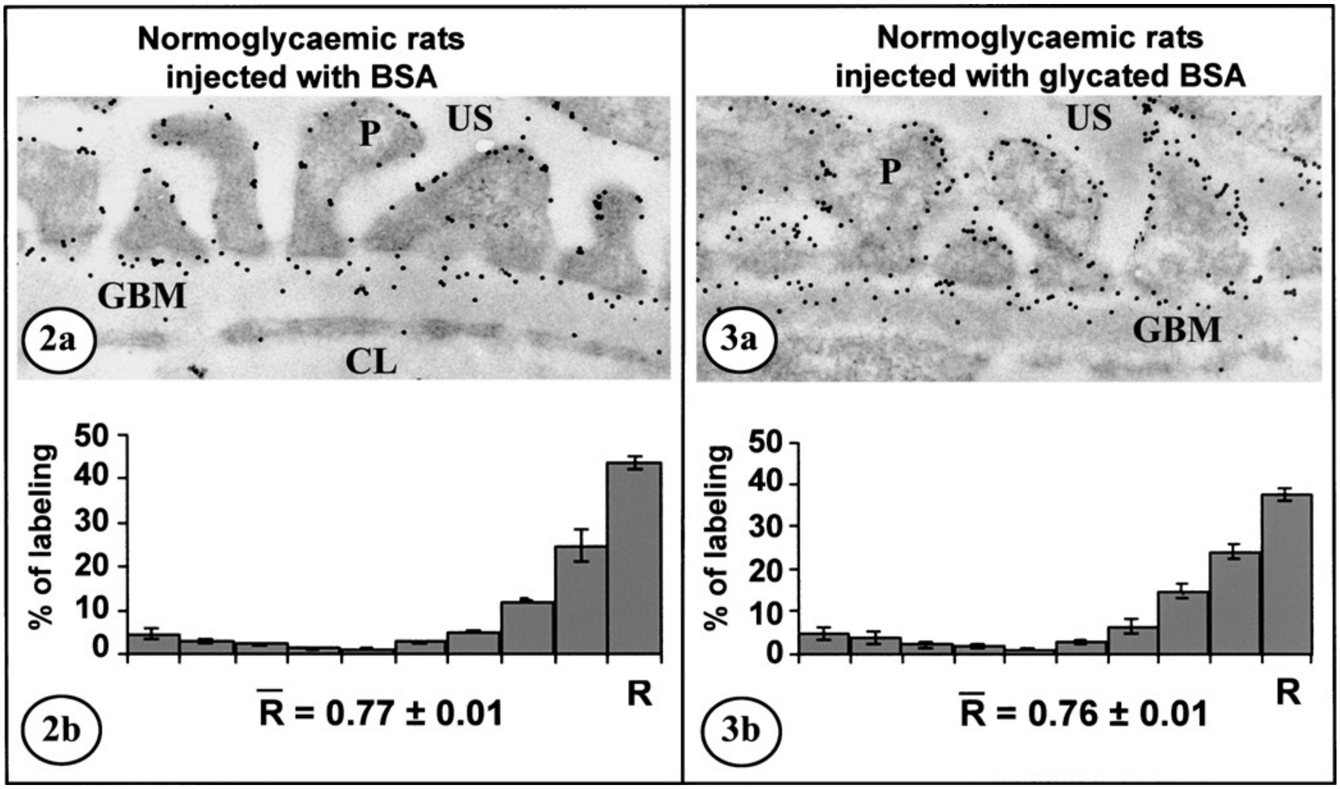

**FIGURES 2 and 3**

Polylysine-gold labeling on the glomerular wall of normal rats injected with either BSA (2*a*) or glycated BSA (3*a*). Over the glomerular basement membrane (GBM), the labeling by gold particles is concentrated on the subepithelial side. Histograms reveal similar GBM labeling distributions in both conditions, with a single peak on the right side, and similar mean *R* values (2*b*, 3*b*). CL, capillary lumen; P, epithelial podocytes; US, urinary space. Polylysine-gold, 10 nm. Magnifications: ×38,000 (2*a*); ×41,000 (3*a*).

on the individual basement membranes along the endothelial and epithelial sides (Figure 8). In more mature glomeruli in the same tissue, displaying fused basement membranes, the GBM labeling was mainly located in the subepithelial area, with few gold particles on the central and subendothelial parts (Figure 9). Histograms obtained through the morphometrical evaluations revealed a rather uniform labeling distribution for the nonfused GBM ($R = 0.56 \pm 0.01$, Figure 8*b*), similar to the histograms obtained in other conditions of altered permeability (Figures 5*b* and 7*b*). A more asymmetrical labeling distribution, with 1 peak on the subepithelial side ($R = 0.64 \pm 0.01$; Figure 8*b*), was obtained for the more mature glomeruli with fused GBM, approaching the situation encountered in young adult normoglycemic animals (Figure 4*b*).

### DISCUSSION

In the present study, the polylysine-gold probe was used for revealing and analyzing the distribution of anionic charges across the GBM of normoglycemic rats infused with glycated albumin, which was reported to alter glomerular permeability. Other related experimental conditions, characterized by increased glomerular permeability, were also evaluated in parallel.

In tissues of normal adult animals, anionic charges are mainly found along the plasma membrane of most renal cells and over all basement membranes. In what concerned the GBM, responsible for the permselectivity of the glomerular wall, charges revealed by the polylysine probe were located in the lamina rara externa, and to a lesser extent in the lamina densa and the lamina rara interna, a pattern similar to that reported with other cationic tracers, such as cuprolinic blue [35]. Based on specific enzymatic treatments, anionic charges recognized by the polylysine-gold correspond to heparan-sulphate proteoglycans in the GBM and to sialoproteins in epithelial and endothelial plasma membranes [31]. Goode and colleagues [10] and Karasawa and colleagues [36] have demonstrated that polylysine-gold complexes also recognize carboxyl groups in chondroitin-sulphate glycosaminoglycans, which have been

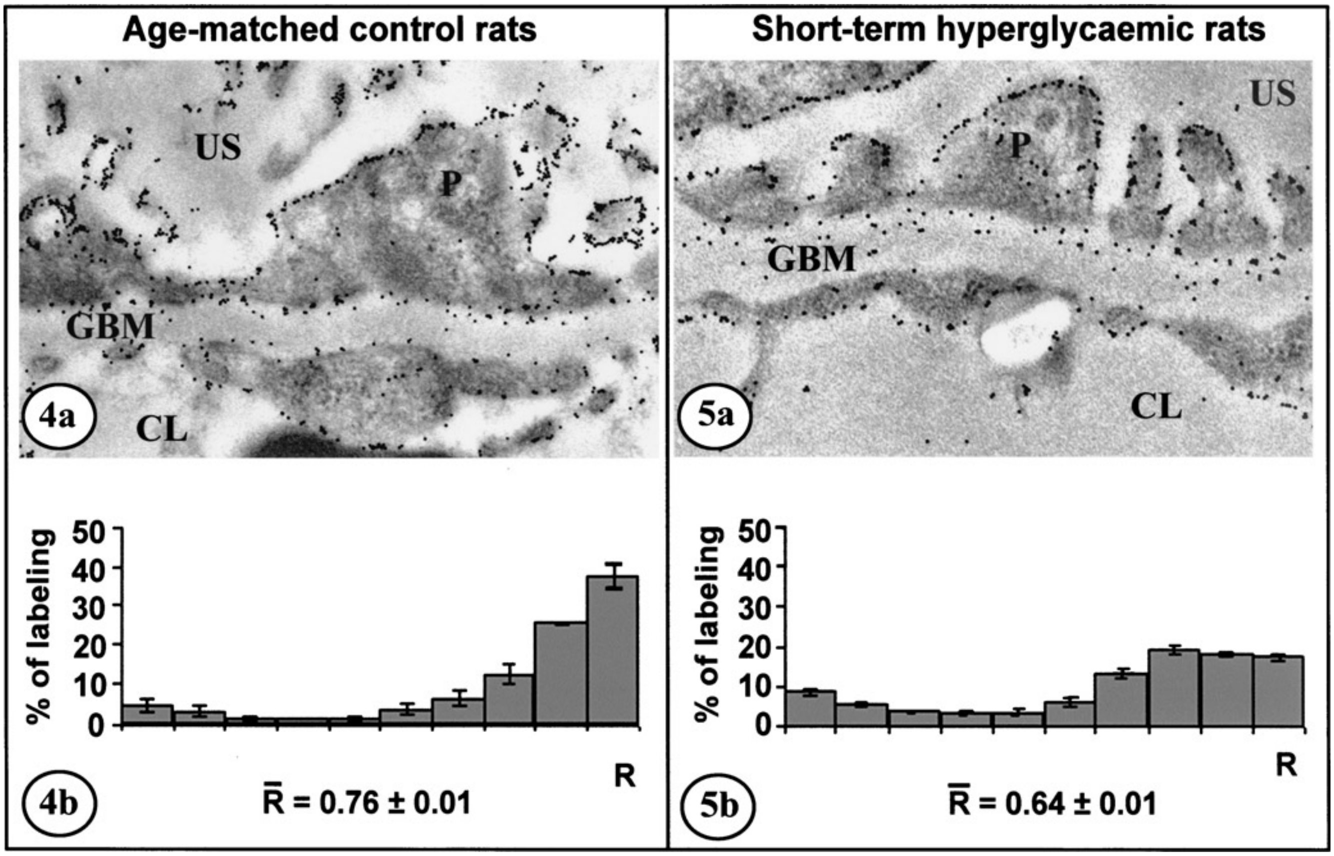

**FIGURES 4 and 5**

Polylysine-gold labeling on the glomerular wall of short-term hyperglycemic rats and their controls. In control rats, labeling over the GBM is mainly located on the subepithelial side, with few particles in the center and the subendothelial side (4*a*). The histogram reveals a single peak of labeling at the right subepithelial side (4*b*). Tissues from the short-term hyperglycemic rats (10 days after streptozotocin injection) present a subepithelial labeling of the GBM, but the gold particles spread in a wider region (5*a*). The histogram displays lower values on the right side of the distribution (5*b*). CL, capillary lumen; P, epithelial podocytes; US, urinary space. Polylysine-gold, 10 nm. Magnification: ×25,000 (4*a*); ×32,000 (5*a*).

shown to contribute in a significant manner to the integrity of the glomerular wall [9].

In previous studies, we as well as others have shown that glycated albumin introduced in the circulation of normal animals can alter the glomerular filtration properties [11, 12, 23]. Introduction of glycated BSA in normal animals induces a transient glomerular alteration that lasts for 48 hours [12]. Changes in glomerular permeability induced by glycated albumin can hardly be due to levels of protein injected because same quantities of native BSA were unable to modify the glomerular permselectivity properties [11]. The albumin injected that traverses the GBM is reabsorbed by proximal tubules and the quantities excreted in urine are too small to be detected or to induce measurable differences. On the other hand, this method was shown to be able to detect changes in GBM permeability [11, 12].

The glycated albumin used in our experiments has been prepared in vitro and contains 1 to 3 modified lysyl residues (fructosyllysine) [11]. This modification renders the protein slightly heavier and more anionic than the native one. However, this modified albumin, once introduced in the circulation of normal animals, penetrates deeper than the GBM and reaches the glomerular urinary space [11], altering also the filtration properties for other circulating molecules [11, 12] that are normally restrained by the lamina rara interna [3]. Surprisingly, in the present study, we found that although major alterations in the glomerular permselectivity took place upon introduction of glycated albumin in circulation, no changes in the anionic charge concentration and distribution across the GBM occur, suggesting that glycated albumin bypass the glomerular permselectivity barrier by mechanisms that do not affect the anionic charges of the GBM.

In contrast, changes in the concentration and distribution of anionic charges were recorded in tissues of long-term diabetic rats that display alterations of the filtration properties. The

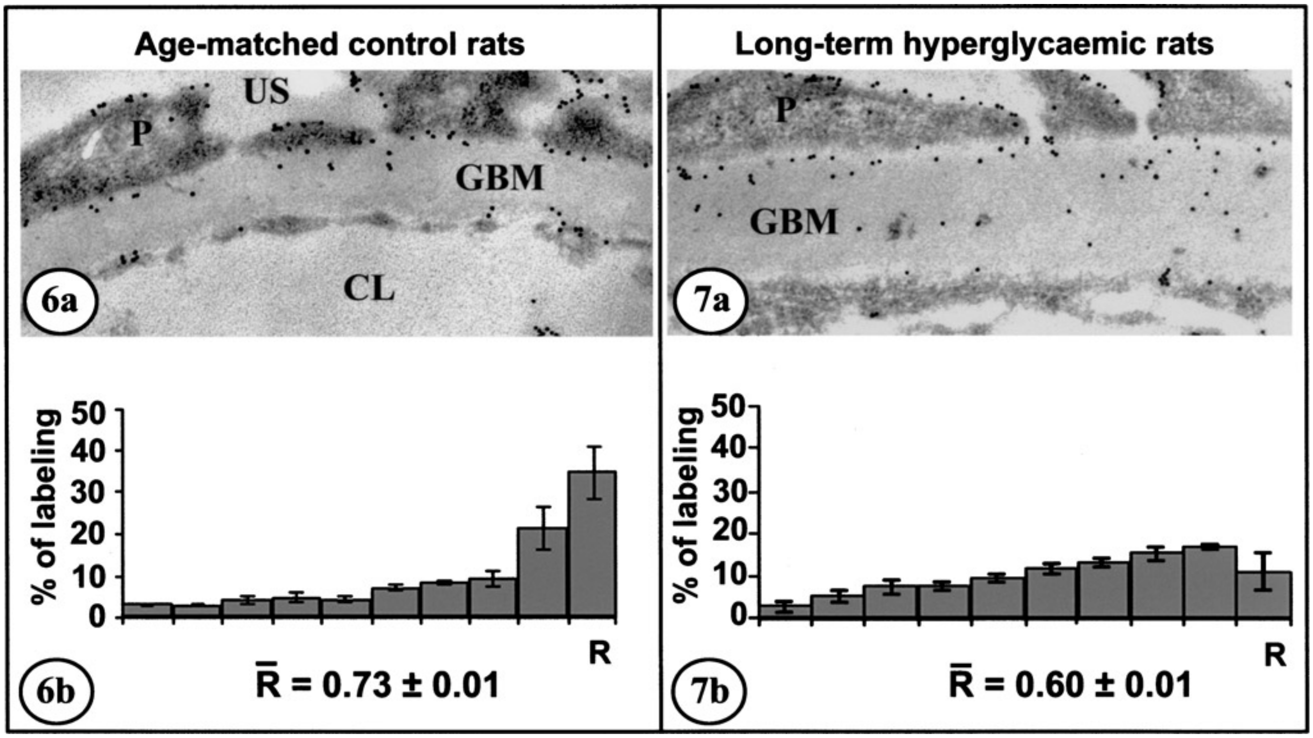

**FIGURES 6 and 7**

Polylysine-gold labeling on the glomerular wall of long-term hyperglycemic rats and their age-matched controls. In old control rats, labeling over the GBM is similar to the one observed in younger animals, with numerous gold particles on the subepithelial side and few throughout the GBM (6*a*). The GBM labeling histogram reveals a single peak at the right subepithelial side (6*b*). Long-term hyperglycemic rats, on the other hand, present a labeling scattered throughout the GBM (7*a*), which yields a rather homogeneous distribution, with low *R* values (7*b*). Notice that GBM thickness is significantly increased in tissues of diabetic animals (7*a*). On the other hand, GBM thickness in normoglycemic animals also varies with age (compare 4*a* with 6*a*). CL, capillary lumen; P, epithelial podocyte; US, urinary space. Polylysine-gold, 15 nm. Magnification: ×32,000 (6*a*); ×36,000 (7*a*).

labeling by polylysine-gold complexes decreased and shifted towards the lamina densa and the lamina rara interna of the GBM. Such alterations in the distribution of the anionic charges parallel the loss of glomerular permselectivity properties. Several parameters can be responsible for these changes, among them the deposition of circulating proteins [37], including glycated products and AGEs [22, 38], and modifications of structural components by glycation [13, 19, 38]. The decrease in the "de novo" synthesis of sulphate-rich proteoglycans in hyperglycemic conditions, the undersulfonation of HSPG, the depolymerization of heparan sulphate, and the generation of reactive oxygen species, have also been suggested as possible mechanisms involved in the pathophysiological events leading to diabetic nephropathy (reviewed in [8]).

In short-term diabetic rats, the alteration in glomerular permeability was demonstrated as early as 10 days after the onset of hyperglycemia [18]. In these rats, the morphological features of the GBM were indistinguishable from those of control animals, although the distribution of anionic charges across the GBM was altered in a pattern similar to that found for the long-term diabetic animals. These results go along with those of Moriya and colleagues [17] and Isogai and colleagues [4] who demonstrated changes in polyethyleneimine binding sites in the GBM of 1- and 2-week hyperglycemic animals, respectively. Thus overall, changes in GBM anionic charge distribution in hyperglycemic animals appear to coincide with impairments in permselectivity properties.

Along the same line, we studied GBM anionic charges in a situation when glomerular permeability is not yet efficiently established. Indeed, the embryonic development of glomerular tissue in 4-day-old rats is not yet fully completed. In such a situation, the distribution of anionic charges was found to be quite different from that of adult animals due to the fact that the assembly of extracellular matrix components, which contributes to the establishment of the permselectivity properties and to the selective restriction of serum proteins, was not completed [33, 39, 40]. By means of cationic dyes, Reeves and colleagues [34] have shown a random distribution of the glycosaminoglycans in

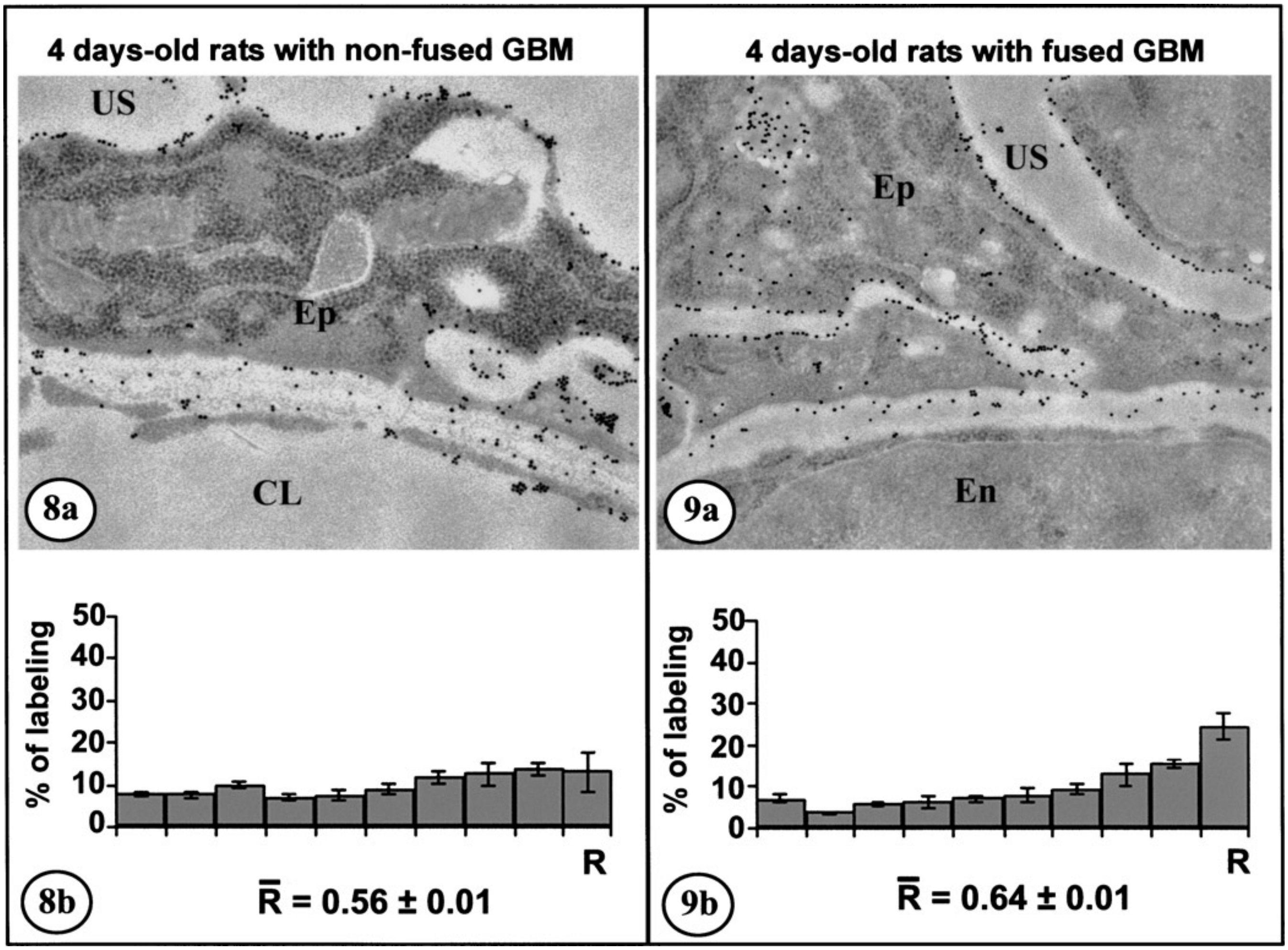

**FIGURES 8 and 9**

Polylysine-gold labeling on glomeruli of 4 days old rats. Labeling is located along the nonfused endothelial and epithelial glomerular basement membranes (8*a*) of immature glomeruli (capillary loop stage) and follows the individual basement membranes. Morphometrical evaluation of this labeling revealed a rather homogenous distribution across the glomerular wall basement membranes (8*b*). In more mature glomeruli of the same kidney, where the basement membranes have fused, the labeling appears more concentrated on the subepithelial side, with some gold particles in the center and subendothelial side (9*a*). The GBM labeling histogram, in this case, shows increasing values towards the right side (9*b*). CL, capillary lumen; En, endothelial cell; Ep, epithelial cell; US, urinary space. Polylysine-gold, 15 nm. Magnification: ×29,000 (8*a*); ×27,000 (9*a*).

the GBM at early developmental stages. Using the polylysine-gold tracer, we have located the anionic charges along the two distinct extracellular matrix layers corresponding to the immature nonfused endothelial and epithelial GBMs. This particular distribution goes along with our results showing that, at this same early stage of maturation, the selective permeability properties of the glomerular wall are not yet established and albumin escapes easily toward the urinary space [33].

These results in newborn and in diabetic animals confirm the importance of the anionic charges in the proper glomerular function and the pertinence of the polylysine-gold probe in revealing their changes. Thus, our experiment with glycated albumin represents a very peculiar situation in which alteration in glomerular permeability occurs while concentration and distribution of the anionic charges remain unchanged.

Because glycated albumin does not seem to affect the anionic charges of the GBM, studies related to the other factors governing the glomerular permselectivity become imminent. Structural changes in the glycated albumin molecule itself have been proposed to explain its altered handling by the GBM [11, 12, 23, 24]. In fact, Shaklai and colleagues [41] have demonstrated that glycation induces conformational changes in serum albumin, concomitant to changes in biological properties. That protein conformation, beside charge and size properties, constitutes a determinant factor for glomerular permselectivity has been established from studies using dextrans [1], Ficoll [42], and proteins with similar Stokes-Einstein radius, but with different physicochemical characteristics [42, 43]. On the other hand, it has also been proposed that glycated proteins could induce the release of mediators that would affect vascular properties [43–45] and likely, glomerular filtration [15].

### REFERENCES

[1] Brenner, B. M., Hostetter, T. H., and Humes, H. D. (1978) Molecular basis of proteinuria of glomerular origin. *N. Engl. J. Med.,* **298,** 826–832.

[2] Remuzzi, A., and Remuzzi, G. (1994) Glomerular permselective function. *Kidney Int.,* **45,** 398–492.

[3] Bendayan, M., Gingras, D., and Charest, P. (1986) Distribution of endogenous albumin in the glomerular wall of streptozotocin-induced diabetic rats as revealed by high-resolution immunocytochemistry. *Diabetologia,* **29,** 868–875.

[4] Isogai, S., Mogami, K., Shiina, N., and Yoshino, G. (1999) Initial ultrastructural changes in pore size and anionic sites of the glomerular basement membrane in streptozotocin-induced diabetic rats and their prevention by insulin treatment. *Nephron,* **83,** 53–58.

[5] Farquhar, M. (1981) The glomerular basement membrane: A selective macromolecular filter. In: *Cell Biology of Extracellular Matrix*, edited by Hay E. D., pp. 335–378. New York, Plenum Press.

[6] Karnovsky, M. J., and Ainsworth, S. K. (1972) The structural basis of glomerular filtration. *Adv. Nephrol. Necker Hosp.,* **2,** 35–60.

[7] Ruotsalainen, V., Ljungberg, P., Wartiovaara, J., Lenkkeri, U., Kestila, M., Jalanko, H., Holmberg, C., and Tryggvason, K. (1999) Nephrin is specifically located at the slit diaphragm of glomerular podocytes. *Proc. Natl. Acad. Sci. U.S.A.,* **96**, 7962–7967.

[8] Raats, C. J. I., Van den Born, J., and Berden, J. H. M. (2000) Glomerular heparan sulfate alterations: Mechanisms and relevance for proteinuria. *Kidney Int.,* **57,** 385–400.

[9] Bertolatus, J. A., and Hunsicker, L. G. (1987) Polycation binding to glomerular basement membrane. Effect of biochemical modification. *Lab. Invest.,* **56,** 170–179.

[10] Goode, N. P., Shires, M., Crellin, D. M., and Davison, A. M. (1991) Detection of glomerular anionic sites in post-embedded ultra-thin sections using cationic colloidal gold. *J. Histochem. Cytochem.,* **39,** 965–972.

[11] Londono, I., Ghitescu, L., and Bendayan, M. (1995) Glomerular handling of circulating glycated albumin in the normal mouse kidney. *Am. J. Physiol.,* **268,** F913–F921.

[12] Londono, I., and Bendayan, M. (2001) Temporary effects of circulating Amadori products on glomerular filtration properties in the normal mouse. *Am. J. Physiol.,* **280,** F103–F111.

[13] Parthasarathy, N., and Spiro, R. G. (1982) Effect of diabetes on the glycosaminoglycan component of the human glomerular basement membrane. *Diabetes,* **31,** 738–741.

[14] Rohrbach, R. (1986) Reduced content and abnormal distribution of anionic sites (acid proteoglycans) in the diabetic glomerular basement membrane. *Virchows Arch. B Cell Pathol.,* **51,** 127–135.

[15] Deckert, T., Feldt-Rasmussen, B., Djurup, R., and Deckert, M. (1988) Glomerular size and charge selectivity in insulin-dependent diabetes mellitus. *Kidney Int.,* **33,** 100–108.

[16] Chakrabarti, S., Ma, N., and Sima, A. A. (1989) Reduced number of anionic sites is associated with glomerular basement membrane thickening in the diabetic BB rat. *Diabetologia,* **32,** 826–828.

[17] Moriya, T., Nakazawa, K., Itoh, N., Shigematsu, H., Okada, N., Aizawa, T., Yamada, T., and Yajima, Y. (1993) Loss of glomerular anionic sites and the development of albuminuria in rats with streptozotocin-induced diabetes. *Nephron,* **65,** 444–448.

[18] Doucet, M., Londono, I., Gomez-Pascual, A., and Bendayan, M. (2000) Glomerular basement membrane selective permeability in short-term streptozotocin-induced diabetic rats. *Int. J. Exp. Diabetes Res.,* **1,** 19–30.

[19] Brownlee, M., and Cerami, A. (1981) The biochemistry of the complications of diabetes mellitus. *Ann. Rev. Biochem.,* **50,** 385–432.

[20] Cohen, M. P., and Ziyadeh, F. N. (1996) Role of Amadori-modified nonenzymatically glycated serum proteins in the pathogenesis of diabetic nephropathy. *J. Am. Soc. Nephrol.,* **7,** 183–190.

[21] Bucala, R., and Vlassara, H. (1995) Advanced glycosylation end products in diabetic renal and vascular disease. *Am. J. Kidney Dis.,* **26,** 875–888.

[22] Bendayan, M. (1998) Immunocytochemical detection of advanced glycated end products in rat renal tissue as a function of age and diabetes. *Kidney Int.,* **54,** 438–447.

[23] Layton, G. J., and Jerums, G. (1988) Effect of glycation of albumin on its renal clearance in normal and diabetic rats. *Kidney Int.,* **33,** 673–676.

[24] Sabbatini, M., Sansone, G., Ucello, F., Giliberti, A., Conte, G., and Andreucci, V. E. (1992) Early glycosylation products induce glomerular hyperfiltration in normal rats. *Kidney Int.,* **42,** 875–881.

[25] Goode, N. P., Shires, M., Crellin, D. M., Aparicio, S. R., and Davison, A. M. (1995) Alterations of glomerular basement membrane charge and structure in diabetic nephropathy. *Diabetologia,* **38,** 1455–1465.

[26] Kanwar, Y. S., and Farquhar, M. G. (1979) Anionic sites in the glomerular basement membrane. In vivo and in vitro localization to the laminae rarae by cationic probes. *J. Cell Biol.,* **81,** 137–153.

[27] Latta, H., Johnston, W. M., and Stanley, T. M. (1975) Sialoglycoproteins and filtration barriers in the glomerular capillary wall. *J. Ultrastr. Res.,* **51,** 354–376.

[28] Michael, A. F., Blau, E., and Vernier, R. L. (1970) Glomerular polyanion: Alteration in aminonucleoside nephrosis. *Lab. Invest.,* **23,** 649–657.

[29] Reale, E., Luciano, L., and Kuhn, K. W. (1983) Ultrastructural architecture of proteoglycans in the glomerular basement membrane. A cytochemical approach. *J. Histochem. Cytochem.,* **31,** 662–668.

[30] Skutelsky, E., and Roth, J. (1986) Cationic colloidal gold—A new probe for the detection of anionic cell surface sites by electron microscopy. *J. Histochem. Cytochem.,* **34,** 693–696.

[31] Russo, P., Gingras, D., and Bendayan, M. (1993) Poly-L-lysine-gold probe for the detection of anionic sites in normal glomeruli and in idiopathic and experimentally-induced nephrosis. A comparative ultrastructural study. *Am. J. Pathol.,* **142,** 261–271.

[32] Bendayan, M. (1995) Colloidal gold post-embedding immunocytochemistry. *Prog. Histochem. Cytochem.,* **29,** 1–163.

[33] Desjardins, M., and Bendayan, M. (1991) Ontogenesis of glomerular basement membrane: Structural and functional properties. *J. Cell Biol.,* **113,** 689–700.

[34] Reeves, W. H., Kanwar, Y. S., and Farquhar, M. G. (1980) Assembly of the glomerular filtration surface. Differentiation of anionic sites in glomerular capillaries of newborn rat kidney. *J. Cell Biol.,* **85,** 735–753.

[35] van den Born, J., van Kraats, A. A., Bakker, M. A., Assmann, K. J., Dijkman, H. B., van der Laak, J. A., and Berden, J. H. (1995) Reduction of heparan sulphate-associated anionic sites in the glomerular basement membrane of rats with streptozotocin-induced diabetic nephropathy. *Diabetologia,* **38,** 1169–1175.

[36] Karasawa, R., Nishi, S., Suzuki, Y., Imai, N., and Arakawa, M. (1997) Early increase of chondroitin sulfate glycosaminoglycan in the glomerular basement membrane of rats with diabetic glomerulopathy. *Nephron,* **76,** 62–71.

[37] Michael, A. F., and Brown, D. M. (1981) Increased concentration of albumin in kidney basement membranes in diabetes mellitus. *Diabetes,* **30,** 843–846.

[38] Brownlee, M., Vlassara, H., and Cerami, A. (1987) The pathogenic role of non-enzymatic glycosylation in diabetic complications. In: *Diabetic Complications. Scientific and Clinical Aspects,* edited by Crabbe, J. C., pp. 94–139. New York: Churchill Livingstone.

[39] Vernier, R. L., and Birch-Anderson, A. (1963) Studies of the human fetal kidney. II. Permeability characteristics of the developing glomerulus. *J. Ultrastr. Res.,* **8,** 66–88.

[40] Bakala, H., Geloso-Meyer, A., Cheignon, M., and Schaeverbeke, J. (1985) Differentiation of the glomerular filtration barrier in the rat fetus: Possible role of collagen. *Connect. Tissue Res.,* **13,** 283–290.

[41] Shaklai, N., Garlick, R. L., and Bunn, H. F. (1984) Nonenzymatic glycosylation of human serum albumin alters its conformation and function. *J. Biol. Chem.,* **259,** 3812–3817.

[42] Ohlson, M., Sorensson, J., Lindstrom, K., Blom, A. M., Fries, E., and Haraldsson, B. (2001) Effects of filtration rate on the glomerular barrier and clearance of four differently shaped molecules. *Am. J. Physiol.,* **281,** F103–F113.

[43] Rennke, H. G., Patel, Y., and Venkatachalam, M. A. (1978) Glomerular filtration of proteins: Clearance of anionic, neutral, and cationic horseradish peroxidase in the rat. *Kidney Int.,* **13,** 278–288.

[44] Amore, A., Cirina, P., Mitola, S., Peruzzi, L., Gianoglio, B., Rabbone, I., Saccheti, C., Cerutti, F., Grillo, C., and Coppo, R. (1997) Nonenzymatically glycated albumin (Amadori adducts) enhances nitric oxide synthase activity and gene expression in endothelial cells. *Kidney Int.,* **51,** 27–35.

[45] Chen, S., Cohen, M. P., Lautenslager, G. T., Shearman, C. W., and Ziyadeh, F. N. (2001) Glycated albumin stimulates TGF-beta 1 production and protein-kinase C activity in glomerular endothelial cells. *Kidney Int.,* **59,** 673–681.

